# Stain Consistency Learning: Handling Stain Variation for Automatic Digital Pathology Segmentation

**DOI:** 10.1109/OJEMB.2026.3687108

**Published:** 2026-04-23

**Authors:** Michael Yeung, Todd Watts, Sean YW Tan, Peiyuan Jing, Pedro F. Ferreira, Andrew D. Scott, Sonia Nielles-Vallespin, Guang Yang

**Affiliations:** Department of ComputingImperial College London 4615 SW7 2AZ London U.K.; Signalling ProgrammeBabraham Institute 2150 CB22 3AT Cambridge U.K.; Department of Clinical NeurosciencesUniversity of Cambridge 2152 CB2 2PY Cambridge U.K.; Department of Bioengineering and Imperial-XImperial College London 4615 W12 7SL London U.K.; National Heart & Lung InstituteImperial College London 4615 SW3 6LY London U.K.; Cardiovascular Magnetic Resonance UnitRoyal Brompton Hospital 156726 SW3 6NP London U.K.; Department of Bioengineering and Imperial-XImperial College London 4615 W12 7SL London U.K.; National Heart and Lung InstituteImperial College London 4615 SW7 2AZ London U.K.; Cardiovascular Research CentreRoyal Brompton Hospital 156726 SW3 6NP London U.K.; School of Biomedical Engineering & Imaging SciencesKings College London 4616 WC2R 2LS London U.K.

**Keywords:** Computational pathology, domain adaptation, instance segmentation, stain augmentation, stain normalization

## Abstract

Stain variation poses a major challenge for automated digital pathology. Numerous techniques address this issue, yet show limited success, especially outside H&E stains and classification tasks. We propose Stain Consistency Learning (SCL), combining stain-specific augmentation and a novel consistency loss to learn stain-invariant features. We conduct the first large-scale evaluation of ten methods on Massons trichrome and H&E datasets for segmentation. Our results demonstrate that traditional stain normalization offers little benefit, while stain augmentation and adversarial learning significantly improve performance. SCL consistently outperforms all other methods.

## I. Introduction

With rising biopsy demands and limited pathologists [1], computational pathology leveraging AI offers efficient interpretation, providing automatic, accurate and efficient interpretation of digital pathology, with performance comparable to expert pathologists [2], [3], [4].

Despite the significant progress made with AI-based computational pathology, there remains several legal, ethical and technical challenges that limit its translation into clinical practice [5]. Among the technical challenges, stain variation represents one of the most important barriers hindering the generalisation of machine learning models to external data. This is because machine learning models may overfit to stain appearances observed on training data, and fail to generalise to images from other centres or scanners which display different staining characteristics [6].

**Fig. 1.**
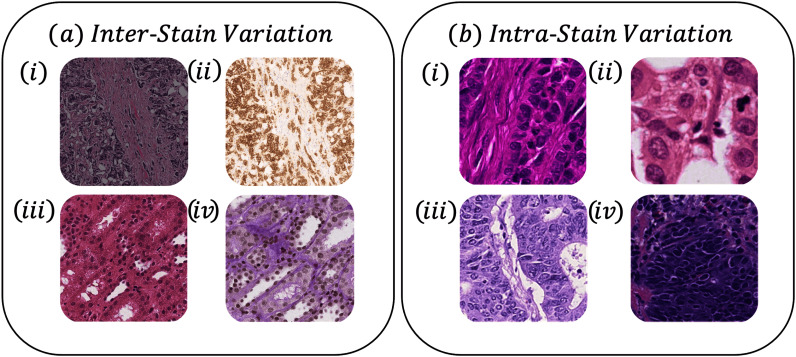
Examples of stain variation in digital pathology. (a) Inter-stain variation of consecutive (i) H&E and (ii) HER2 receptor antibody stained data, and (iii) H&E and (iv) Periodic acidSchiff (PAS) stained data. b) Intra-stain variation of H&E stained data. Inter-stain variation images were selected from Breast Cancer Immunohistochemical (BCI) dataset (a iii) and Center for Applied Medical Research (CIMA) histology dataset (a iiiiv), and intra-stain variation images were selected from the MIDOG dataset (b iii) and Lizard dataset (b iiiiv).

Stains are chemical dyes applied to histology slides to enhance tissue contrast and highlight certain structures. The most widely used stain is haematoxylin and eosin (H&E), which stains nuclei dark purple and the extracellular matrix and cytoplasm pink. For specific situations, other stains may be preferred, such as Massons trichrome stain for connective tissue, or immunohistochemical stains for proteins. Depending on the stain used, the same tissue section can differ considerably in appearance (Fig. 1).

Alongside inter-stain variation, there is also intra-stain variation, where tissue appearances may differ despite applying the same stain. Stain variation arises during the complex process of acquiring digital pathology, where differences in tissue preparation protocol, stain manufacturer and scanner type may contribute towards both inter-stain and intra-stain variation [7].

**Fig. 2.**
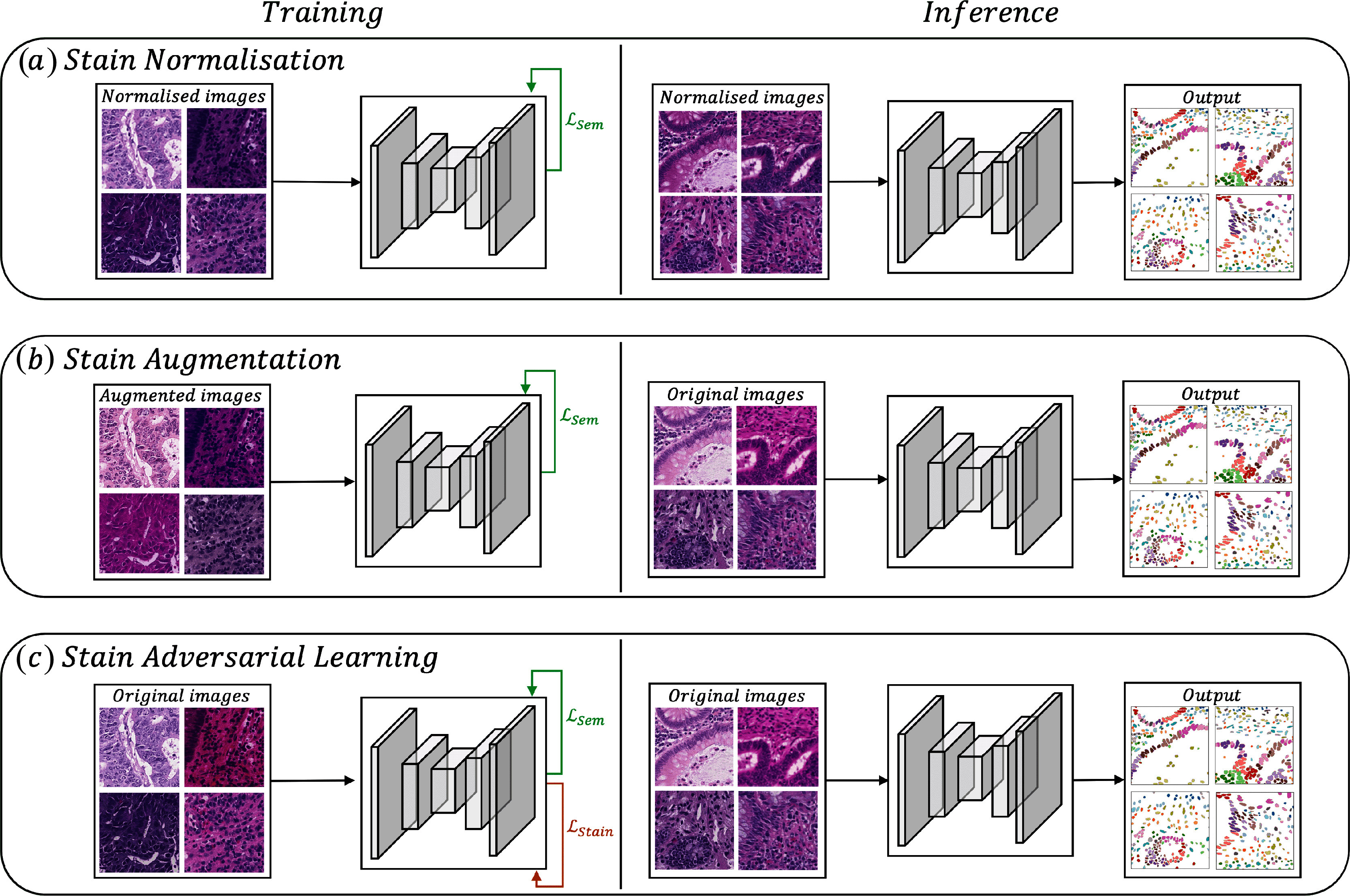
Overview of methods to handle stain variation on digital pathology. (a) Stain normalisation methods standardise the staining prior to training and inference, removing the need for machine learning models to learn stain variation. (b) Stain augmentation methods apply stain-specific perturbations to the training data, increasing the diversity of staining observed by the model during training. (c) Stain adversarial learning involves joint optimisation of a specialised loss function ($\mathcal {L}_{\mathrm{Stain}}$) to learn stain-invariant features in addition to the downstream task ($\mathcal {L}_{\mathrm{Sem}}$).

In recent years, there has been considerable progress in developing methods to improve the robustness of machine learning models to stain variation [7], [8]. These methods can be broadly categorised into stain normalisation, stain augmentation and stain adversarial learning approaches (Fig. 2).

Stain normalisation handles stain variation by standardising the staining prior to model training and inference, reducing the total variation in staining seen by the model. Broadly, stain normalisation approaches can be divided into statistical, stain separation and deep learning methods. Statistical methods involve matching the image characteristics of a reference image to a set of images and include histogram matching (HM) [9], [10], Fourier Domain Adaptation (FDA) [11], [12], [13], and Reinhards method [14]. These methods can be applied to a wide variety of imaging data but are prone to generating artefacts or require careful hyperparameter tuning. Stain separation methods were developed specifically for histology data, and involve matching the stain characteristics of a reference image to a set of images. Ruifrok and Johnston proposed the first stain separation method, known as the colour deconvolution (CD) algorithm [15], which decomposes an image into stain colour and stain concentration components. However, CD is not scalable because it requires obtaining the stain colour matrix empirically. Later approaches focused on automatically extracting the stain colour matrix through matrix decomposition, including the use of singular value decomposition (SVD) [16], non-negative matrix factorisation (NMF) [17], [18] and integrated optimisation [19]. In recent years, deep learning-based methods using generative adversarial networks (GANs) have become a popular choice for performing stain normalisation, reframing the problem as an unsupervised, style-transfer task [20], [21], [22], [23]. There has been progress in reducing the inference time [23], as well as adapting GANs to handle multiple domains [24], [25]. However, it is currently unclear whether GANs are suitable methods for clinical use because there are uncertainties as to whether the original image content could be altered during the transformation [26].

While stain normalisation must be applied during inference, stain augmentation and stain adversarial approaches are only necessary during training. Stain augmentations are colour augmentation methods that were developed for histology images [27], [28], [29]. Stain Jitter generates diversity by applying noise directly to the H&E colour channels extracted using CD [27], with two hyperparameters controlling the degree of pertubation. RandStainNA is a more recent approach that overcomes the need for hyperparameter tuning by using the training data to determine the degree of stain perturbation [29]. The use of GANs to generate stain diversity has also been explored [28], [30], [31], although existing GAN-based augmentation approaches are computationally expensive and require domain labels for training [32].

Stain adversarial methods encourage optimisation for learning stain-invariant features using specialised loss functions. Domain-adversarial neural network (DANN) training involves the use of a task-specific loss, such as classification or segmentation, together with a domain classification loss [33]. To avoid the need for domain labels, Marini et al. replaced the domain classification loss with a regression loss and trained models to maximise the regression loss when predicting H&E colour matrices [34]. However, stain adversarial methods rely on network modifications for generating auxiliary outputs, limiting their flexibility for integration into existing pipelines.

Despite numerous methods developed to handle stain variation, many of the methods have not been widely adopted due to problems associated with computational complexity, need for domain labels or hyperparameter tuning, and concern over image content alterations. Moreover, the majority of methods to handle stain variation were developed and evaluated on classification tasks using H&E-stained data and it is unclear whether these methods generalise to different stains or tasks. To address these issues, we propose a novel framework, named stain consistency learning (SCL), to handle variation of different stains for segmentation tasks. In this paper, we propose the following contributions:

1) We propose stain consistency learning, a novel method that incorporates stain-specific augmentation with a specialised loss function to encourage the learning of stain colour invariant features.
2) We perform the first large-scale comparison of methods to handle stain variation for segmentation tasks, evaluating ten methods on datasets of Massons trichrome stained cells and H&E stained nuclei, and show that stain consistency learning outperforms all other methods across datasets.
3) We demonstrate the benefit of leveraging larger, unlabelled datasets to improve the performance of stain augmentation methods, alleviating the dependency on stain variation within the training data.

## II. Materials and Methods

### A. Stain Consistency Learning

SCL incorporates a new stain augmentation method to increase stain diversity observed by the model, and a complementary loss function to encourage the learning of stain colour invariant features.

#### 1) Stain Consistency Augmentation

**Fig. 3.**
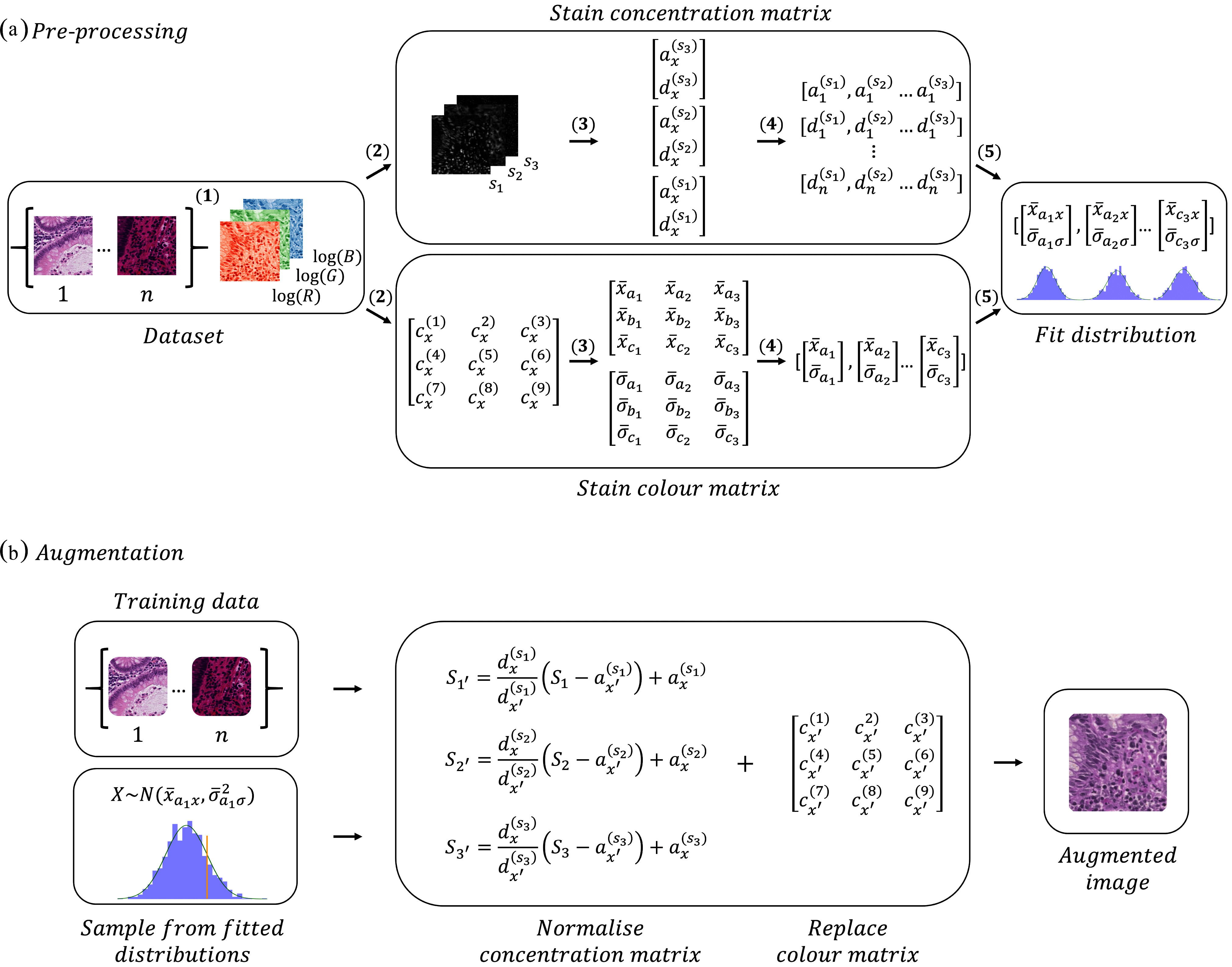
Overview of Stain Consistency Augmentation. (a) Pre-processing. (1) Each image is first converted into optical density space. (2) Images are decomposed into a stain colour and stain concentration matrix using Macenkos method. (3) The mean and standard deviation statistics are extracted per channel for the stain concentration matrix, and per element for the stain colour matrix. (4) Mean and standard deviation statistics are aggregated across the dataset. (5) A normal distribution is fit for each statistic. (b) Augmentation. During training, the selected image is decomposed into stain colour and stain concentration matrices. The stain colour matrix is replaced with one sampled from the fitted distributions, while the per-channel mean and standard deviation statistics of the stain concentration matrix are normalised using the sampled stain concentration values, generating an image with augmented stain colour and concentration properties.

Unlike previous approaches relying on a single reference matrix, SCA (Fig. 3) independently extracts a stain color matrix from each image independently.

To augment an image (I), the image is first converted to optical density (OD) space:

\begin{equation*}
\mathrm{I}_{OD}= -\log _{10} (\mathrm{I}). \tag{1}
\end{equation*}

In OD space, the image can be decomposed into a stain colour matrix (**C**) and stain concentration matrix (**S**):

\begin{equation*}
\mathrm{I}_{OD}= \mathbf {C}\mathbf {S}. \tag{2}
\end{equation*}

Macenkos method was used to obtain the stain colour matrix [16] due to its favorable trade-off between accuracy and efficiency, although the proposed framework is not restricted to this choice of stain separation method. The stain concentration matrix can then be obtained by inverting the stain colour matrix:

\begin{equation*}
\mathbf {S} = \mathbf {C}^{-1} \mathrm{I}_{OD}. \tag{3}
\end{equation*}

For each image, we compute the stain colour matrix $\mathbf {C}_{i} \in \mathbb{R}^{3 \times 3}$, as well as the mean $\mathbf {A}_{i}=[a_{i}^{(s_{1})}, a_{i}^{(s_{2})}, a_{i}^{(s_{3})}] \in \mathbb{R}^{3}$ and standard deviations $\mathbf {D}_{i}=[d_{i}^{(s_{1})}, d_{i}^{(s_{2})}, d_{i}^{(s_{3})}] \in \mathbb{R}^{3}$ for each stain $s \in \lbrace s_{1}, s_{2}, s_{3} \rbrace$ in the stain concentration matrix.

Following this, we adopt RandStainNAs approach by fitting a distribution over the dataset for the extracted features [29]. Specifically, we fit three multivariate Gaussian distributions $F_{C}$, $F_{A}$, and $F_{D}$, for the stain colour matrix, as well as the mean and standard deviation of the stain concentration matrix, respectively:

\begin{align*}
 F_{C} \sim \mathcal {N}(\boldsymbol{M}_{C}, \boldsymbol{\Sigma }_{C}), \\
F_{A} \sim \mathcal {N}(\boldsymbol{M}_{A}, \boldsymbol{\Sigma }_{A}), \\
F_{D} \sim \mathcal {N}(\boldsymbol{M}_{D}, \boldsymbol{\Sigma }_{D}). \tag{4}
\end{align*}

To augment a given image $\mathrm{I}_{x}$, we first extract the stain colour matrix $\mathbf {C}_{x} \in \mathbb{R}^{3 \times 3}$, as well as the per-channel mean $\mathbf {A}_{x}=[a_{x}^{(s_{1})}, a_{x}^{(s_{2})}, a_{x}^{(s_{3})}]$ and standard deviation $\mathbf {D}_{x}=[d_{x}^{(s_{1})}, d_{x}^{(s_{2})}, d_{x}^{(s_{3})}] \in \mathbb{R}^{3}$ of the stain concentration matrix. Next, we sample from the fitted distributions to obtain a stain colour matrix $\mathbf {C}_{x^{\prime }} \in \mathbb{R}^{3 \times 3}$, and sample mean $\mathbf {A}_{x^{\prime }}=[a_{x^{\prime }}^{(s_{1})}, a_{x^{\prime }}^{(s_{2})}, a_{x^{\prime }}^{(s_{3})}]$ and standard deviations $\mathbf {D}_{x^{\prime }}=[d_{x^{\prime }}^{(s_{1})}, d_{x^{\prime }}^{(s_{2})}, d_{x^{\prime }}^{(s_{3})}] \in \mathbb{R}^{3}$ to normalise the stain concentration matrix. We replace the stain colour matrix $\mathbf {C}_{x}$ with $\mathbf {C}_{x^{\prime }}$, and transform the stain concentration matrix $\mathbf {S}$:

\begin{align*}
 S_{1^{\prime }} & =\frac{d_{x}^{(s_{1})}}{d_{x^{\prime }}^{(s_{1})}}\left(s_{1}-a_{x^{\prime }}^{(s_{1})}\right)+a_{x}^{(s_{1})} \\
S_{2^{\prime }} & =\frac{d_{x}^{(s_{2})}}{d_{x^{\prime }}^{(s_{2})}}\left(s_{2}-a_{x^{\prime }}^{(s_{2})}\right)+a_{x}^{(s_{2})} \\
S_{3^{\prime }} & =\frac{d_{x}^{(s_{3})}}{d_{x^{\prime }}^{(s_{3})}}\left(s_{3}-a_{x^{\prime }}^{(s_{3})}\right)+a_{x}^{(s_{3})}. \tag{5}
\end{align*}

Finally, the modified stain colour and stain concentration matrices are combined and converted to RGB space, producing the augmented image, $\mathrm{I}_{x^{\prime }}$:

\begin{equation*}
\mathrm{I}_{x^{\prime }}= e^{-\mathbf {C}_{x^{\prime }}\mathbf {S}_{x^{\prime }}}. \tag{6}
\end{equation*}

#### 2) Stain Consistency Loss

**Fig. 4.**
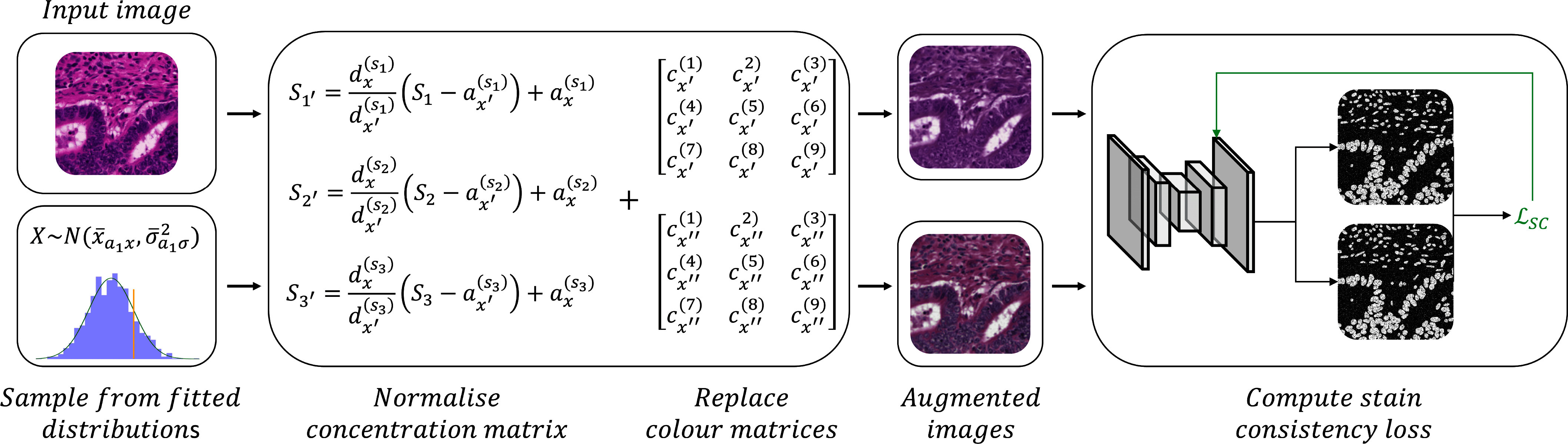
Overview of Stain Consistency Learning. For each training image, we sample a single stain concentration matrix and two stain colour matrices from our fitted distributions. These are used to generate two augmented variants, by normalising the original image with the same stain concentration matrix and replacing the stain colour matrix with different stain colour matrices. Both augmented variants are then input into the model separately, and the stain consistency loss is computed based on the two segmentation outputs.

Stain augmentation methods encourage the learning of stain-invariant features by requiring model predictions to remain accurate in the presence of stain variation. To bridge the gap between stain augmentation and stain adversarial learning approaches, we instead propose SCL (Fig. 4).

SCL optimizes models to learn features invariant to stain color while remaining sensitive to concentration differences. Two augmented versions of the same image, differing only in color matrices but sharing concentration information, are input during training. Their segmentation outputs are compared using a mean absolute error loss, excluding background pixels. By passing both images as inputs, we define the stain consistency loss ($\mathcal {L}_{\mathrm{SC}}$) as the mean absolute error between the two segmentation outputs, $p$ and $p^{\prime }$, excluding background pixels and following transformation using a sigmoid activation function:

\begin{equation*}
\mathcal {L}_{\mathrm{SC}}(p, p^{\prime })= \frac{1}{m} \sum _{i \in M} |p_{i} - p^{\prime }_{i}|, \tag{7}
\end{equation*}

where m iterates over the pixels corresponding to the ground truth objects.

### B. Dataset Description

#### 1) Lizard Dataset

The Lizard dataset is the largest open-source dataset with instance segmentation labels in digital pathology, comprised of 495,179 labeled nuclei from six sources [35]. We use the training image patches of the Lizard dataset prepared by the CoNIC challenge [36] (Table IV, available online).

The Lizard dataset consists of H&E-stained whole slide images of colonic tissue at 20 objective magnification. Nuclei instance segmentation masks were generated through a semi-automatic approach, using HoVer-Net to provide annotations that were subsequently manually refined for challenging cases [37]. The CoNIC challenge provides 4,981 256256 non-overlapping patches, from which we remove all patches without nuclei, leaving 4,841 patches for training and evaluation.

#### 2) Cardiomyocyte Dataset

Our in-house dataset consists of four porcine hearts that were subsequently formalin fixed and paraffin embedded [38]. Transmural sections were obtained at 5 $\mu$m thickness and stained with Massons trichrome. We obtained high-resolution microscopy data at 20 magnification with 456 nm/pixel resolution (C9600-12, Hamamatsu, Hamamatsu City, Japan). We randomly extracted 200 224 224 patches per heart, with two sets of cell instance segmentation labels separately provided by two individuals experienced with histology data.

### C. Implementation Details

We used the VersaTile^1^^1^VersaTile: A Framework for Instance Segmentation. Available at: https://github.com/TissueImageAnalytics/VersaTile. framework for instance segmentation, with the default settings. The data augmentation settings for VersaTile are shown in Table V (available online). For cell segmentation, we perform four-fold cross validation, involving successive training on images from one subject, and evaluation on images from the other three subjects. For the Lizard dataset, we use image patches from DigestPath for training, and image patches from CoNSeP, CRAG, GlaS and PanNuke for evaluation. We selected DigestPath for training because it is the only dataset within the Lizard dataset that does not have an overlapping dataset source with the other datasets. For each method, we train three models, initialised with different random seeds, and report the average performance. All experiments were conducted on NVIDIA RTX-6000 GPU. For evaluating, we use two popular instance segmentation metrics: F1 and PQ score.

## III. Results

### A. Qualitative Comparison of Stain Augmentation Methods

**Fig. 5.**
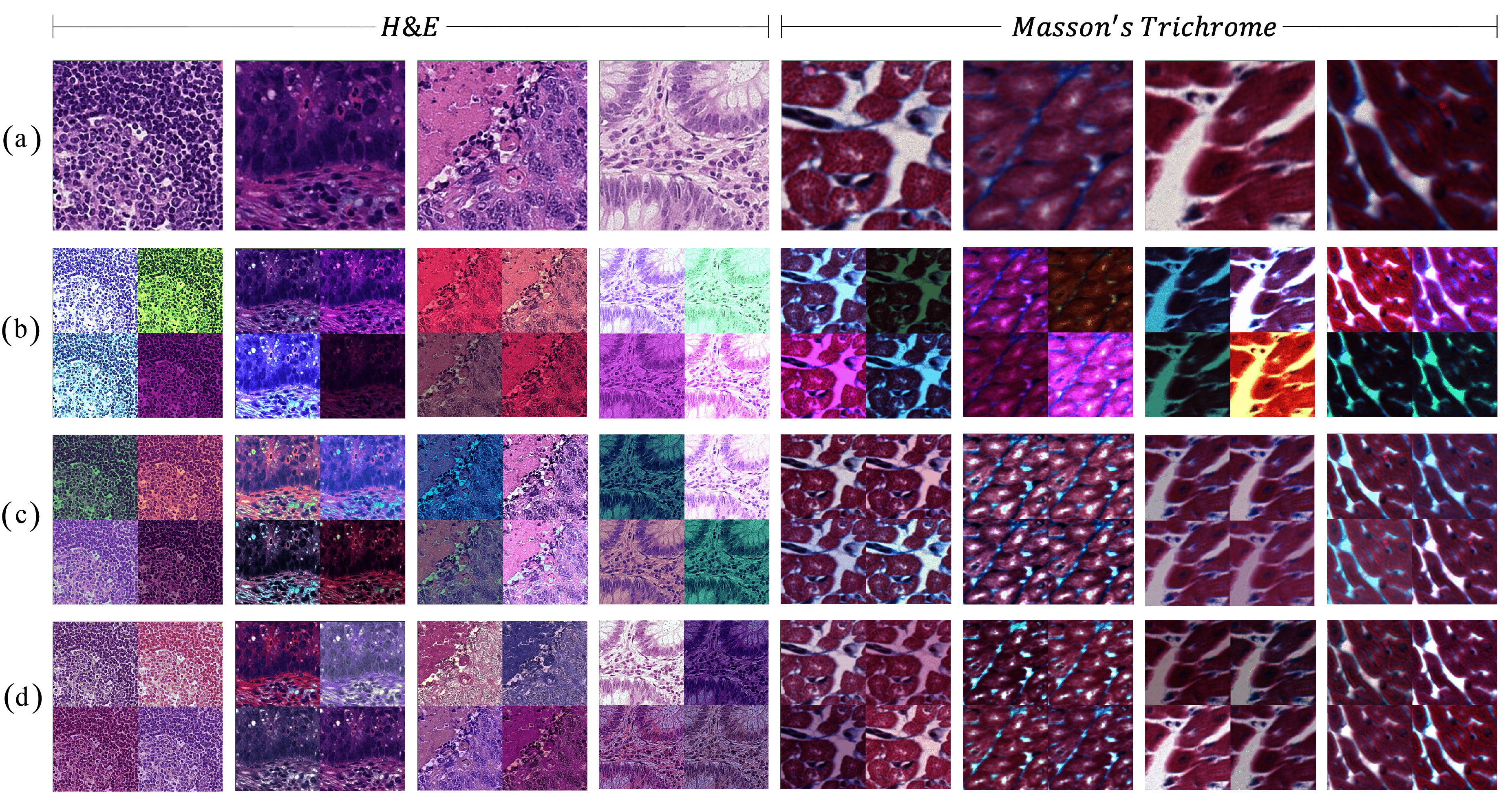
Example images after applying different stain augmentation methods. (a) Original image. (b) Stain Jitter [27]. (c) RandStainNA ($L\alpha \beta$ space) [29]. (d) SCA (proposed). The images were selected from the Lizard dataset and our in-house dataset to represent a range of stain appearances. Each augmented image demonstrates four consecutive applications of the stain augmentation method on the original image. RandStainNA and SCA parameters were fit on the Lizard dataset. For Stain Jitter, we set $\alpha$ = 0.25 and $\beta$ = 0.05 [27].

To provide a qualitative comparison of different stain augmentation methods, we provide a range of examples of augmented H&E images from the Lizard dataset and Massons trichrome images from our in-house dataset in Fig. 5. The appearance of stain normalised images are available in the supplementary materials (Fig. 6).

As shown in Fig. 5, stain augmentation methods are able to generate a diverse range of stain appearances. While Stain Jitter appears to produce the most diverse range of colour variation, it also generates the largest number of unrealistic augmented images. This is particularly evident with poorly stained images, where the addition of noise could result in stain colour values shifting to outside of the realistic range. In contrast, both RandStainNA and SCA do not display systematic errors and maintain generally realistic stain variation across the sample of images. However, applying RandStainNA occasionally results in the introduction of colour artefacts, which is not apparent with SCA, and overall SCA generates the most realistic stain variation.

### B. Quantitative Comparison on Instance Segmentation

We evaluate the effects of using various stain normalisation, stain augmentation and stain adversarial learning methods on cell and nuclei segmentation performance.

The results for cell segmentation are shown in Table I.

**TABLE I:** Performance on Cell Segmentation Using Different Methods to Handle Stain Variation. The Best Performance is Denoted in Bold. The Bootstrapped 95 Confidence Intervals are Shown in Brackets. The Overall Performance is Computed as the Mean Performance Using Annotator 1 and 2 as Ground Truth Labels.

	Annotator 1		Annotator 2		Overall	
Method	$F1_{50}$ ($\uparrow$)	$PQ_{50}$ ($\uparrow$)	$F1_{50}$ ($\uparrow$)	$PQ_{50}$ ($\uparrow$)	$F1_{50}$ ($\uparrow$)	$PQ_{50}$ ($\uparrow$)
Baseline	0.735 ($\pm$0.005)	0.576 ($\pm$0.005)	0.736 ($\pm$0.005)	0.586 ($\pm$0.005)	0.736 ($\pm$0.005)	0.581 ($\pm$0.005)
HM [10]	0.725 ($\pm$0.005)	0.564 ($\pm$0.005)	0.723 ($\pm$0.005)	0.572 ($\pm$0.005)	0.724 ($\pm$0.005)	0.568 ($\pm$0.005)
FDA [11]	0.729 ($\pm$0.005)	0.568 ($\pm$0.005)	0.729 ($\pm$0.005)	0.579 ($\pm$0.005)	0.729 ($\pm$0.005)	0.574 ($\pm$0.005)
Reinhard et al. [14]	0.734 ($\pm$0.005)	0.573 ($\pm$0.005)	0.736 ($\pm$0.005)	0.584 ($\pm$0.005)	0.735 ($\pm$0.005)	0.579 ($\pm$0.005)
Macenko et al. [16]	0.723 ($\pm$0.005)	0.566 ($\pm$0.005)	0.715 ($\pm$0.005)	0.561 ($\pm$0.005)	0.719 ($\pm$0.005)	0.564 ($\pm$0.005)
SPCN [18]	0.722 ($\pm$0.005)	0.560 ($\pm$0.005)	0.722 ($\pm$0.005)	0.567 ($\pm$0.005)	0.722 ($\pm$0.005)	0.564 ($\pm$0.005)
ACD [19]	0.732 ($\pm$0.005)	0.573 ($\pm$0.005)	0.729 ($\pm$0.005)	0.579 ($\pm$0.005)	0.731 ($\pm$0.005)	0.576 ($\pm$0.005)
Stain Jitter [27]	0.739 ($\pm$0.005)	0.580 ($\pm$0.005)	0.737 ($\pm$0.005)	0.587 ($\pm$0.005)	0.738 ($\pm$0.005)	0.584 ($\pm$0.005)
RandStainNA [29]	0.739 ($\pm$0.005)	0.580 ($\pm$0.005)	0.741 ($\pm$0.005)	0.591 ($\pm$0.005)	0.740 ($\pm$0.005)	0.586 ($\pm$0.005)
SCA (proposed)	0.745 ($\pm$0.005)	0.586 ($\pm$0.005)	0.745 ($\pm$0.005)	0.595 ($\pm$0.005)	0.745 ($\pm$0.005)	0.591 ($\pm$0.005)
SCL (proposed)	**0.754 ($\pm$0.005)**	**0.594 ($\pm$0.005)**	**0.757 ($\pm$0.005)**	**0.606 ($\pm$0.005)**	**0.756 ($\pm$0.005)**	**0.600 ($\pm$0.005)**

Overall, the best performing method was SCL, with an F1 score of 0.756 ($\pm$0.005) and PQ score of 0.600 ($\pm$0.005). This was followed by SCA, with an overall F1 score of 0.745 ($\pm$0.005) and PQ score of 0.591 ($\pm$0.005). All stain augmentation methods performed significantly better than the baseline for both F1 scores (Stain Jitter: $p={9.2e-9}$, RandStainNA: $p={0.017}$, SCA: $p={9.2e-9}$, SCL: $p< {1e-10}$) and PQ scores (Stain Jitter: $p={3.1e-10}$, RandStainNA: $p={5.0e-3}$, SCA: $p={3.1e-10}$, SCL: $p< {1e-10}$). In contrast, stain normalisation approaches were associated with equivalent F1 (Reinhard: $p={0.18}$, SPCN: $p={0.12}$) and PQ (Reinhard: $p={0.12}$) scores, or worse F1 (HM: $p={2.9e-3}$, FDA: $p={0.014}$, Macenko: $p={0.0014}$, ACD: $p={2.4e-7}$) and PQ (HM: $p={1.8e-3}$, FDA: $p={0.0077}$, Macenko: $p={5.3e-5}$, SPCN: $p={0.0023}$, ACD: $p={1.8e-9}$) scores.

The results for nuclei segmentation are shown in Table II.

**TABLE II:** Performance on Nuclei Segmentation Using Different Methods to Handle Stain Variation. The Best Performance is Denoted in Bold. The Bootstrapped 95 Confidence Intervals are Shown in Brackets. The Overall Performance is the Mean Performance Across the Four Datasets.

	CoNSeP		CRAG		GlaS		PanNuke		Overall	
Method	$F1_{50}$ ($\uparrow$)	$PQ_{50}$ ($\uparrow$)	$F1_{50}$ ($\uparrow$)	$PQ_{50}$ ($\uparrow$)	$F1_{50}$ ($\uparrow$)	$PQ_{50}$ ($\uparrow$)	$F1_{50}$ ($\uparrow$)	$PQ_{50}$ ($\uparrow$)	$F1_{50}$ ($\uparrow$)	$PQ_{50}$ ($\uparrow$)
Baseline	0.671 ($\pm$0.010)	0.505 ($\pm$0.008)	0.614 ($\pm$0.003)	0.471 ($\pm$0.002)	0.691 ($\pm$0.003)	0.531 ($\pm$0.003)	0.711 ($\pm$0.009)	0.539 ($\pm$0.007)	0.672 ($\pm$0.006)	0.512 ($\pm$0.005)
HM [10]	0.645 ($\pm$0.012)	0.479 ($\pm$0.010)	0.567 ($\pm$0.003)	0.430 ($\pm$0.003)	0.668 ($\pm$0.004)	0.507 ($\pm$0.003)	0.676 ($\pm$0.011)	0.511 ($\pm$0.009)	0.639 ($\pm$0.008)	0.482 ($\pm$0.006)
FDA [11]	0.597 ($\pm$0.015)	0.438 ($\pm$0.011)	0.506 ($\pm$0.004)	0.382 ($\pm$0.003)	0.657 ($\pm$0.004)	0.496 ($\pm$0.003)	0.624 ($\pm$0.013)	0.465 ($\pm$0.01)	0.596 ($\pm$0.009)	0.445 ($\pm$0.007)
Reinhard et al. [14]	0.646 ($\pm$0.011)	0.482 ($\pm$0.009)	0.603 ($\pm$0.003)	0.460 ($\pm$0.002)	0.666 ($\pm$0.004)	0.510 ($\pm$0.003)	0.692 ($\pm$0.009)	0.522 ($\pm$0.007)	0.652 ($\pm$0.007)	0.494 ($\pm$0.005)
Macenko et al. [16]	0.631 ($\pm$0.013)	0.472 ($\pm$0.010)	0.518 ($\pm$0.004)	0.391 ($\pm$0.003)	0.665 ($\pm$0.003)	0.503 ($\pm$0.003)	0.674 ($\pm$0.011)	0.507 ($\pm$0.009)	0.622 ($\pm$0.008)	0.468 ($\pm$0.006)
SPCN [18]	0.613 ($\pm$0.013)	0.458 ($\pm$0.010)	0.522 ($\pm$0.004)	0.394 ($\pm$0.003)	0.667 ($\pm$0.004)	0.504 ($\pm$0.003)	0.640 ($\pm$0.013)	0.477 ($\pm$0.010)	0.611 ($\pm$0.009)	0.458 ($\pm$0.007)
ACD [19]	0.644 ($\pm$0.013)	0.484 ($\pm$0.010)	0.527 ($\pm$0.004)	0.403 ($\pm$0.003)	0.702 ($\pm$0.003)	0.538 ($\pm$0.003)	0.691 ($\pm$0.011)	0.523 ($\pm$0.009)	0.641 ($\pm$0.008)	0.487 ($\pm$0.006)
StainGAN [22]	0.500 ($\pm$0.016)	0.362 ($\pm$0.012)	0.552 ($\pm$0.003)	0.414 ($\pm$0.002)	0.540 ($\pm$0.004)	0.388 ($\pm$0.003)	0.568 ($\pm$0.013)	0.420 ($\pm$0.010)	0.540 ($\pm$0.009)	0.396 ($\pm$0.007)
Stain Jitter [27]	0.692 ($\pm$0.009)	0.521 ($\pm$0.008)	0.677 ($\pm$0.002)	0.518 ($\pm$0.002)	0.704 ($\pm$0.003)	0.530 ($\pm$0.003)	0.720 ($\pm$0.007)	0.547 ($\pm$0.006)	0.698 ($\pm$0.005)	0.529 ($\pm$0.005)
RandStainNA [29]	0.682 ($\pm$0.009)	0.513 ($\pm$0.008)	0.613 ($\pm$0.003)	0.469 ($\pm$0.002)	0.677 ($\pm$0.003)	0.520 ($\pm$0.003)	0.715 ($\pm$0.008)	0.543 ($\pm$0.007)	0.672 ($\pm$0.006)	0.511 ($\pm$0.005)
H&E Adversarial [34]	0.680 ($\pm$0.010)	0.509 ($\pm$0.008)	0.639 ($\pm$0.003)	0.489 ($\pm$0.002)	0.683 ($\pm$0.003)	0.524 ($\pm$0.003)	0.719 ($\pm$0.009)	0.546 ($\pm$0.007)	0.680 ($\pm$0.006)	0.517 ($\pm$0.005)
SCA (proposed)	0.695 ($\pm$0.010)	0.524 ($\pm$0.008)	**0.685 ($\pm$0.002)**	**0.526 ($\pm$0.002)**	0.719 ($\pm$0.003)	0.545 ($\pm$0.003)	0.723 ($\pm$0.008)	0.549 ($\pm$0.007)	0.706 ($\pm$0.006)	0.536 ($\pm$0.005)
SCL (proposed)	**0.696 ($\pm$0.010)**	**0.525 ($\pm$0.008)**	0.683 ($\pm$0.002)	0.524 ($\pm$0.002)	**0.726 ($\pm$0.003)**	**0.553 ($\pm$0.002)**	**0.730 ($\pm$0.007)**	**0.555 ($\pm$0.007)**	**0.709 ($\pm$0.006)**	**0.539 ($\pm$0.005)**

Similar to cell segmentation, SCL was the best performing method for nuclei segmentation, with an overall F1 score of 0.709 ($\pm$0.006) and PQ score of 0.539 ($\pm$0.005). This was followed by SCA with an overall F1 score of 0.706 ($\pm$0.006) and PQ score of 0.536 ($\pm$0.006). Both stain augmentation and stain adversarial methods performed significantly better than the baseline for both F1 and PQ scores (all $p< {1e-10}$). In contrast, all stain normalisation methods had significantly lower overall F1 and PQ scores (all $p< {1e-10}$).

### C. Effect of Pre-Processing Datasets on Stain Augmentation

Both RandStainNA and SCA involve learning a distribution over the images in the training data to generate stain diversity. Rather than using the training data, it is possible to learn the stain distribution using a larger unlabelled dataset, which would allow models to learn stain variation not present in the training data. To evaluate the effect of using different datasets for pre-processing on segmentation performance, we pre-process RandStainNA and SCA on the MIDOG dataset and the whole Lizard dataset [13]. The results are shown in Table III.

**TABLE III:** Performance on Nuclei Segmentation Using Different Pre-Processing Datasets for Stain Augmentation Methods. DigestPath is the Training Data Used in the Other Experiments and is Shown for Reference. The Best Performances are Denoted in Bold. The Bootstrapped 95 Confidence Intervals are Shown in Brackets. The Overall Performance is Computed as the Mean Performance Across the Four Datasets.

		CoNSeP		CRAG		GlaS		PanNuke		Overall	
Method	Dataset	$F1_{50}$ ($\uparrow$)	$PQ_{50}$ ($\uparrow$)	$F1_{50}$ ($\uparrow$)	$PQ_{50}$ ($\uparrow$)	$F1_{50}$ ($\uparrow$)	$PQ_{50}$	$F1_{50}$ ($\uparrow$)	$PQ_{50}$ ($\uparrow$)	$F1_{50}$ ($\uparrow$)	$PQ_{50}$ ($\uparrow$)
RandStainNA	DigestPath	0.682 ($\pm$0.009)	0.513 ($\pm$0.008)	0.613 ($\pm$0.003)	0.469 ($\pm$0.002)	0.677 ($\pm$0.003)	0.520 ($\pm$0.003)	0.715 ($\pm$0.008)	0.543 ($\pm$0.007)	0.672 ($\pm$0.006)	0.511 ($\pm$0.005)
SCA (proposed)	DigestPath	0.695 ($\pm$0.010)	0.524 ($\pm$0.008)	**0.685 ($\pm$0.002)**	**0.526 ($\pm$0.002)**	0.719 ($\pm$0.003)	0.545 ($\pm$0.003)	0.723 ($\pm$0.008)	0.549 ($\pm$0.007)	0.706 ($\pm$0.006)	0.536 ($\pm$0.005)
SCL (proposed)	DigestPath	**0.696 ($\pm$0.010)**	**0.525 ($\pm$0.008)**	0.683 ($\pm$0.002)	0.524 ($\pm$0.002)	**0.726 ($\pm$0.003)**	**0.553 ($\pm$0.002)**	**0.730 ($\pm$0.007)**	**0.555 ($\pm$0.007)**	**0.709 ($\pm$0.006)**	**0.539 ($\pm$0.005)**
RandStainNA	MIDOG	0.676 ($\pm$0.010)	0.508 ($\pm$0.008)	0.625 ($\pm$0.003)	0.479 ($\pm$0.002)	0.685 ($\pm$0.003)	0.527 ($\pm$0.003)	0.708 ($\pm$0.009)	0.538 ($\pm$0.008)	0.674 ($\pm$0.006)	0.513 ($\pm$0.005)
SCA (proposed)	MIDOG	0.693 ($\pm$0.011)	0.523 ($\pm$0.009)	**0.700 ($\pm$0.002)**	**0.537 ($\pm$0.002)**	0.715 ($\pm$0.003)	0.540 ($\pm$0.003)	**0.726 ($\pm$0.008)**	**0.551 ($\pm$0.007)**	0.709 ($\pm$0.006)	0.538 ($\pm$0.005)
SCL (proposed)	MIDOG	**0.696 ($\pm$0.011)**	**0.524 ($\pm$0.009)**	0.695 ($\pm$0.002)	0.533 ($\pm$0.002)	**0.723 ($\pm$0.003)**	**0.552 ($\pm$0.003)**	0.725 ($\pm$0.007)	**0.551 ($\pm$0.007)**	**0.710 ($\pm$0.006)**	**0.540 ($\pm$0.005)**
RandStainNA	Lizard	0.680 ($\pm$0.010)	0.512 ($\pm$0.008)	0.608 ($\pm$0.003)	0.466 ($\pm$0.002)	0.677 ($\pm$0.003)	0.521 ($\pm$0.003)	**0.719 ($\pm$0.008)**	**0.545 ($\pm$0.007)**	0.671 ($\pm$0.006)	0.511 ($\pm$0.005)
SCA (proposed)	Lizard	**0.697 ($\pm$0.011)**	**0.525 ($\pm$0.009)**	**0.696 ($\pm$0.002)**	**0.535 ($\pm$0.002)**	0.726 ($\pm$0.003)	0.554 ($\pm$0.003)	0.715 ($\pm$0.010)	0.544 ($\pm$0.008)	**0.709 ($\pm$0.007)**	**0.540 ($\pm$0.006)**
SCL (proposed)	Lizard	0.692 ($\pm$0.010)	0.519 ($\pm$0.009)	0.692 ($\pm$0.002)	0.531 ($\pm$0.002)	**0.727 ($\pm$0.009)**	**0.555 ($\pm$0.002)**	0.715 ($\pm$0.011)	0.543 ($\pm$0.007)	0.707 ($\pm$0.008)	0.537 ($\pm$0.005)

Overall, we observed better segmentation performance using images from the MIDOG or Lizard dataset for stain augmentation pre-processing, compared to using images from the training data. SCA performance significantly improved from an F1 score of 0.706 ($\pm$0.006) and PQ score of 0.536 ($\pm$0.005), to an F1 score of 0.709 ($\pm$0.007) and PQ score of 0.540 ($\pm$0.006), when using the Lizard dataset for pre-processing ($p< {1e-10}$). Specifically, the CRAG subset was associated with the greatest performance improvement, where the F1 score improved from 0.685 ($\pm$0.002) to 0.696 ($\pm$0.002), and PQ score improved from 0.526 ($\pm$0.002) to 0.535 ($\pm$0.002). Less consistent performance improvements were observed using SCL and RandStainNA. The best overall performance was associated with SCL pre-processed on MIDOG, with an F1 score improvement to 0.710 ($\pm$0.006) ($p={0.072}$) and PQ score improvement to 0.540 ($\pm$0.005) ($p={1.9e-4}$).

## IV. Discussion

Instance segmentation is a challenging task requiring fine-grained, pixel-level understanding, and with significant clinical applications such as cell counting, morphological abnormality detection and quantitative biomarker analysis. Through extensive evaluations, we found that stain augmentation and adversarial learning significantly outperform stain normalization methods on instance segmentation tasks, aligning with prior multi-center studies on classification tasks [8].

We proposed a novel stain augmentation method named SCA that extends upon the methods of Stain Jitter and RandStainNA. Rather than using a single, empirically determined stain colour matrix, we used Macenkos method to extract individual stain colour matrices for each image. We leveraged the diversity in stain colour matrices to generate realistic stain-specific variation (Fig. 5), and observed performance benefits over other methods to handle stain variation across Massons trichrome-stained cardiomyocytes (Table I) and H&E-stained nuclei segmentation (Table II). Realistic stain augmentation is crucial to avoid corrupting clinically important information and to facilitate disentanglement of meaningful stain information from stain color variation introduced by stain protocol differences. Instead of using the training data for preprocessing, we explored learning the stain distribution from external data and observed performance benefits (Table III). This provides a convenient mechanism to overcome limited stain diversity in the training data by learning the stain distribution on a larger, more diverse dataset, without the need for labels.

To integrate stain augmentation and stain adversarial learning for handling stain variation, we developed stain consistency learning, a framework that encourages the model to learn stain colour invariant features while preserving sensitivity to stain concentration differences. This method demonstrated the best performance overall across both datasets (Tables I and II).

Alongside performance, SCL offers several advantages over prior methods. Firstly, neither the proposed augmentation nor loss function require hyperparameter tuning, unlike stain augmentation approaches such as Stain Jitter, which has two hyperparameters [27], and stain normalisation approaches such ACD, which has five hyperparameters [19]. Secondly, SCL is applied only during training, avoiding inference-time overhead and structural changes to the images. Thirdly, SCL does not require modifications to the neural network architecture and can therefore be more easily integrated into existing frameworks, in contrast to stain adversarial approaches which require an additional auxiliary output. Finally, in the era of foundation models, dataset size and diversity are central to achieve robust generalisation, and SCL is well suited for integration into such training pipelines, scaling naturally with dataset size and improving stain diversity for underrepresented stains.

For future work, there are several areas where SCL can be extended. Firstly, dataset balancing methods could further improve the learning of a distribution that represents diverse stain appearances. Secondly, it would be useful to evaluate our framework on other image recognition tasks, to determine whether our method can also provide performance benefits to other tasks. Finally, we demonstrated that SCL improves robustness to intra-stain variation, but it would be useful to extend this framework to also handle inter-stain variation.

## V. Conclusion

We proposed Stain Consistency Learning (SCL), a novel framework that combines stain-specific augmentation with a consistency loss to address stain variation in digital pathology segmentation. Through the first large-scale comparison of ten stain-handling methods across Massons trichrome and H&E datasets, SCL consistently achieved superior performance. We also demonstrated that leveraging larger unlabeled datasets further enhances robustness. SCL offers practical advantages: no hyperparameter tuning, no inference-time cost, and seamless integration into existing models. Future work will explore extending SCL to address inter-stain variation and broader digital pathology tasks, paving the way for more robust and generalizable AI systems in clinical practice.

## Supplementary Materials

Supplementary Materials
