# Supplementary material for: Stain Consistency Learning: Handling Stain Variation for Automatic Digital Pathology Segmentation
Source: IEEE Open J Eng Med Biol. 2026 Apr 23;7:187–95. doi: 10.1109/OJEMB.2026.3687108 (PMC13278751; doi:10.1109/OJEMB.2026.3687108)
Supplement: Supplementary Materials [file supp1-3687108.pdf]

Supplementary Materials

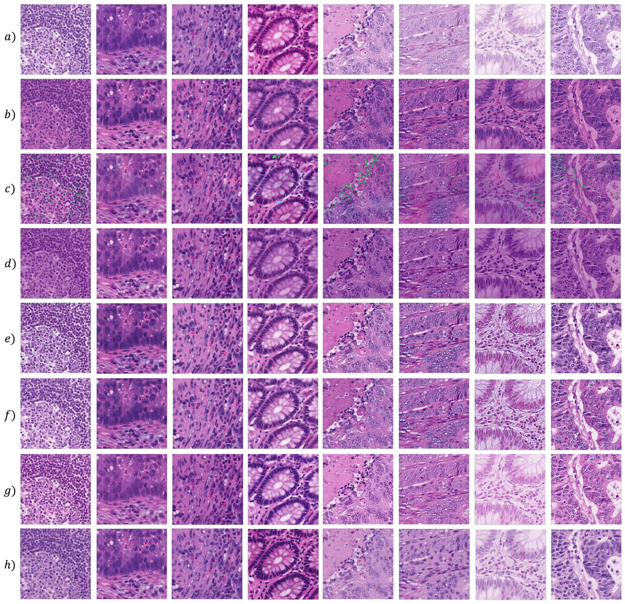

Fig. 1. Example images after applying different stain normalisation methods. a) Original image. b) HM. c) FDA. d) Reinhard et al. e) Macenko et al. f) SPCN. g) ACD. h) StainGAN. The images were carefully selected from the Lizard dataset to represent a range of stain appearances. Where applicable, normalised images used the same reference image.

TABLE I. Lizard dataset sources. All datasets except for DigestPath contain data from University Hospitals Coventry and Warwickshire (UHCW). The sixth dataset source in Lizard, The Cancer Genome Atlas Program (TCGA), was not included in the Colon Nuclei Identification and Counting Challenge (CoNIC) challenge training data.

**Dataset**	**Source**	**#Images**	**#Annotations**
DigestPath	Multiple centres in China	1,788	225,859
CoNSeP	UHCW	64	6,595
CRAG	UHCW	2,181	210,957
GlaS	UHCW	698	112,292
PanNuke	Multiple centres in USA and UHCW	110	14,158

TABLE II. Data augmentation settings. All augmentations used the default settings in Albumentations version 1.3.1. One of CLAHE, RandomGamma and RandomBrightnessContrast was used per augmentation.

**Augmentation**	**Setting**
RandomResizedCrop	scale=(0.08, 1), ratio=(0.75, 1.3), p=0.25
HorizontalFlip	p=0.5
VerticalFlip	p=0.5
RandomRotation90	p=0.5
CLAHE	p=0.25
RandomGamma	gamma_limit=(80,120), p=0.25
RandomBrightnessContrast	brightness_limit=0.2, contrast_limit=0.2, p=0.25
Blur	blur_limit=7, p=0.25
